# Retrospective Evaluation of L-Acetyl Carnitine and Palmitoylethanolamide as Add-On Therapy in Patients with Fibromyalgia and Small Fiber Neuropathy

**DOI:** 10.3390/pharmaceutics17081004

**Published:** 2025-07-31

**Authors:** Crescenzio Bentivenga, Arrigo Francesco Giuseppe Cicero, Federica Fogacci, Natalia Evangelia Politi, Antonio Di Micoli, Eugenio Roberto Cosentino, Paolo Gionchetti, Claudio Borghi

**Affiliations:** 1 Cardiovascular Medicine Unit, Heart, Thoracic and Vascular Department, IRCCS Azienda Ospedaliero-Universitaria di Bologna, 40100 Bologna, Italy; arrigo.cicero@unibo.it (A.F.G.C.); antonio.dimicoli@aosp.bo.it (A.D.M.); eugenio.cosentino@aosp.bo.it (E.R.C.); claudio.borghi@unibo.it (C.B.); 2 Hypertension and Cardiovascular Risk Research Center, Medical and Surgical Sciences Department, Alma Mater Studiorum University of Bologna, 40100 Bologna, Italy; federica.fogacci@studio.unibo.it; 3 Italian Society of Nutraceuticals (SINut), 40100 Bologna, Italy; 4 Department of Medical and Surgical and Sciences, University of Bologna, 40100 Bologna, Italy; natalia.politi2@unibo.it (N.E.P.); paolo.gionchetti@unibo.it (P.G.); 5 BD Unit, IRCCS Azienda Ospedaliero-Universitaria di Bologna, 40100 Bologna, Italy

**Keywords:** fibromyalgia, small fiber neuropathy, L-Acetyl carnitine, palmitoylethanolamide, add-on therapy, retrospective study, fibromyalgia impact questionnaire

## Abstract

Fibromyalgia is a complex disorder characterized by chronic widespread pain and a variety of related symptoms. Growing evidence suggests that the central and peripheral nervous systems are involved, with small fiber neuropathy playing a key role in its development. We retrospectively reviewed the medical records of 100 patients diagnosed with primary fibromyalgia. Those showing symptoms indicative of small fiber dysfunction who were treated with L-Acetyl Carnitine (LAC) and Palmitoylethanolamide (PEA) alongside standard care (SOC) were compared to matched controls who received only SOC. To ensure comparable groups, propensity score matching was used. Changes in Fibromyalgia Impact Questionnaire Revised (FIQR) scores over 12 weeks were analyzed using non-parametric tests due to the data’s non-normal distribution. After matching, 86 patients (43 in each group) were included. The group receiving LAC and PEA as add-on therapy experienced a significant median reduction in FIQR scores (−19.0 points, *p* < 0.001), while the SOC-only group showed no significant change. Comparisons between groups confirmed that the improvement was significantly greater in the LAC+PEA group (*p* < 0.001). These results suggest that adding LAC and PEA to standard care may provide meaningful symptom relief for fibromyalgia patients with suspected small fiber involvement. This supports the hypothesis that peripheral nervous system dysfunction contributes to the disease burden in this subgroup. However, further prospective controlled studies are needed to confirm these promising findings.

## 1. Introduction

Fibromyalgia syndrome (FM) is a complex and debated disorder characterized primarily by widespread chronic pain, often localized in the musculoskeletal system, accompanied by various nonspecific clinical manifestations involving multiple organ systems [1]. Common symptoms include sleep disturbances, intense asthenia and fatigue, neurovegetative alterations (such as gastrointestinal and genitourinary dysfunctions, dizziness, tinnitus, and tachycardia), as well as psychological symptoms like anxiety, panic attacks, depression, and cognitive impairments including memory and attention deficits, obtundation, and confusion [2]. Collectively, these symptoms contribute to a significantly reduced quality of life in affected individuals [1,2].

Despite extensive research, the etiopathogenesis of FM remains incompletely understood [2,3]. Emerging clinical evidence from structural and functional neuroimaging, electrophysiological studies, and skin biopsy analyses suggests involvement of both the central and peripheral nervous systems in FM pathogenesis. In particular, dysfunction in nociceptive afferent processing appears to be a key mechanism underlying FM physiopathology.

Additionally, increasing attention has been drawn to the role of small fiber neuropathy (SFN), which is frequently observed in FM patients undergoing skin biopsy [1,2,3,4]. SFN is characterized by selective or predominant impairment of small myelinated Aδ fibers and unmyelinated C fibers, which are responsible for transmitting sensations such as temperature, pain, and pruritus, as well as mediating sudomotor and autonomic functions related to gastrointestinal, genitourinary, cardiovascular, and thermoregulatory systems. In FM, clinical manifestations of SFN are heterogeneous, with common presentations including limb polyneuropathy and non-limb-related mononeuropathy. Although skin biopsy remains the gold standard for SFN diagnosis, its pathological findings are common among FM patients, and the extent of small fiber involvement correlates with symptom severity [5,6].

L-Acetyl Carnitine (LAC) exhibits antioxidant and anti-inflammatory properties and exerts beneficial effects on neuropathic pain by modulating neurotransmitters such as glutamate and GABA, promoting nerve regeneration, and enhancing synaptic plasticity [7]. Palmitoylethanolamide (PEA), on the other hand, modulates inflammation and pain by activating cannabinoid receptors and inhibiting inflammatory mediators within nerves [8,9]. Recent studies have evaluated a fixed combination of PEA and LAC, suggesting that this supplement provides additional benefits when combined with standard of care (SOC) in patients with secondary rheumatic neuropathies [10].

Furthermore, multiple lines of evidence support the efficacy of LAC and PEA in managing FM symptoms, demonstrating improvements in pain control, psychiatric symptoms, and quality of life [11,12,13,14]. A recent randomized clinical trial investigated the incremental benefit of PEA plus LAC as an add-on therapy to duloxetine and pregabalin in a heterogeneous cohort of FM patients, reporting superior outcomes in terms of widespread pain (assessed via the Widespread Pain Index) and disease burden (measured by the Fibromyalgia Impact Questionnaire Revised, FIQR) [15].

In this context, we conducted a comparative retrospective study to evaluate the efficacy of combined LAC and PEA supplementation as add-on therapy to standard treatment in patients with fibromyalgia showing clinical signs of SFN. These patients were compared to fibromyalgia patients without apparent peripheral symptoms who had received SOC alone. The primary outcome was the variation in Fibromyalgia Impact Questionnaire Revised (FIQR) scores, analyzed both between and within groups, from baseline to 3-month follow-up, using data retrospectively collected from routine clinical practice.

## 2. Patients and Methods

### 2.1. Study Dedign

This retrospective study included the last 100 patients who met inclusion and exclusion criteria and were referred to our outpatient clinic before 31 January 2023. Patients were divided into two cohorts based on clinical presentation and prior treatment. The treatment cohort (*n* = 50) received, in addition to SOC, a fixed combination of LAC (500 mg) and PEA (600 mg) administered twice daily for eight weeks and once daily during the final week. This was delivered as part of the nutraceutical formulation Kalanit^®^ (also containing Boswellia serrata extract, vitamin B6, and vitamin E). The control cohort (*n* = 50) consisted of patients treated exclusively with SOC.

Inclusion required completion of the Italian version of the FIQR questionnaire at treatment initiation and after approximately 12 weeks (±2 weeks). The primary endpoint was the within- and between-group change in FIQR scores over this period.

The study involved the retrospective evaluation of anonymized clinical data collected during standard care, in full compliance with institutional regulations. It was conducted in accordance with the principles of the Declaration of Helsinki and was approved by the Institutional Review Board (IRB) of AOU Sant’Orsola-Malpighi (Bologna, Italy). Given the retrospective design and the use of anonymized data, the requirement for informed consent was waived.

### 2.2. Study Population

The retrospective analysis included 100 patients aged >18 years, with no sex restrictions, who were referred to the cardiovascular risk outpatient clinics of Rheumatology and Cardiovascular Internal Medicine at AOU Sant’Orsola-Malpighi. All patients fulfilled the 2016 American College of Rheumatology (ACR) criteria for the diagnosis of primary fibromyalgia [16], and completed the Italian version of the FIQR at baseline and after approximately 12 weeks (±2 weeks).

Patients were divided into two cohorts based on clinical presentation. The treatment cohort included individuals exhibiting symptoms suggestive of SFN, such as thermo-pain disturbances, paresthesia, pruritus, electric-shock-like sensations, and sudomotor abnormalities. SFN was presumed based on these clinical signs, as no objective diagnostic testing (e.g., skin biopsy or QST) was performed, consistent with the retrospective and real-world setting of the study. Exclusion criteria included current treatment with anticonvulsants (gabapentin, pregabalin, valproate, topiramate), use of cannabinoids or illicit drugs, and comorbidities potentially confounding SFN symptoms, such as diabetes, rheumatic diseases (e.g., Sjögren’s syndrome, vasculitis), malignancies, vitamin or electrolyte deficiencies, decompensated endocrine, cardiovascular, hematologic, or pulmonary diseases, neurological or psychiatric conditions, or recent SARS-CoV-2 infection (<3 months).

At baseline, all patients were receiving SOC for fibromyalgia, comprising pharmacological treatments (antidepressants, muscle relaxants, analgesics) combined with non-pharmacological interventions such as behavioral modifications, relaxation techniques, aerobic and stretching exercises (sometimes in thermal settings or with heating pads), and cognitive-behavioral therapy [17].

### 2.3. Statistical Analysis

All statistical analyses were conducted using SPSS version 26.0 (IBM^®^). Due to the non-normal distribution of FIQR scores, as confirmed via the Shapiro–Wilk test, non-parametric methods were employed. Within-group comparisons of FIQR scores before and after treatment were performed using the Wilcoxon signed-rank test, while between-group comparisons of score changes were conducted using the Mann–Whitney U test.

To minimize baseline differences and potential selection bias between treatment groups, propensity score matching (PSM) was applied. Propensity scores were estimated via logistic regression based on age, sex, baseline FIQR score, and disease duration. Patients in the treatment and control groups were matched 1:1 using nearest neighbor matching without replacement and a caliper width of 0.2 standard deviations of the logit of the propensity score. All analyses were performed on the matched dataset using complete cases only.

A two-tailed *p*-value of less than 0.05 was considered statistically significant, with 95% confidence interval (CI).

## 3. Results

A retrospective analysis was conducted on 100 patients (90% female) aged between 27 and 85 years (mean age 58.87 ± 10.49 years), with a disease duration ranging from 1 to 20 years (mean 6.25 ± 3.68 years). All patients were receiving treatment with a combination of an antidepressant and a muscle relaxant. The distribution of sex was balanced between the two cohorts, with 46 women in the group receiving the add-on LAC and PEA treatment, and 44 women in the group treated with SOC alone. Patients with peripheral symptoms showed a higher baseline disease burden (mean FIQR 74.64 ± 8.24) compared to those without such symptoms (56.78 ± 11.92).

After propensity score matching, 86 patients (43 per group) were retained for the final analysis, ensuring balanced baseline characteristics, including age, sex, baseline FIQR score, and disease duration. The matched cohort consisted of 88.4% female patients, with a mean age of 58.6 ± 10.2 years and a mean disease duration of 6.1 ± 3.4 years.

At baseline, the median FIQR score in the group receiving LAC and PEA as add-on therapy was 73.0 (IQR: 69.0–78.0), while in the SOC group it was 72.0 (IQR: 68.0–76.0), with no statistically significant difference (*p* = 0.41).

After 12 weeks of treatment, patients in the LAC+PEA group demonstrated a significant reduction in FIQR scores, with a median change of −19.0 points (IQR: −25.0 to −13.0; *p* < 0.001, Wilcoxon signed-rank test). In contrast, the SOC-only group showed a marginal, non-significant median change of −1.0 point (IQR: −6.0 to +3.0; *p* = 0.12). The between-group comparison of change in FIQR scores revealed a statistically significant difference favoring the LAC+PEA group (*p* < 0.001, Mann–Whitney U test).

These findings indicate a clinically meaningful and statistically significant improvement in fibromyalgia-related symptoms among patients treated with LAC and PEA, compared to those receiving standard care alone.

## 4. Discussion

Fibromyalgia is a syndrome characterized by chronic widespread pain, sleep disturbances, fatigue, and cognitive impairment, all of which profoundly impact quality of life [16,17]. SFN, on the other hand, affects the thinnest peripheral nerve fibers responsible for transmitting pain and temperature signals to the brain. Dysfunction of these somatosensitive fibers can cause spontaneous chronic pain even in the absence of external stimuli. Moreover, these fibers play a crucial role in regulating blood pressure, heart rate, body temperature, and gastrointestinal and urinary functions [18,19].

Numerous studies have demonstrated a loss of peripheral small fibers in approximately 50% of fibromyalgia patients [20], and those with small fiber damage tend to experience more severe symptoms and a poorer quality of life [6]. Small fiber pathology may contribute to neurogenic microvasculopathy, which could explain several fibromyalgia manifestations, such as skeletal muscle perfusion deficits, deep pain, exercise intolerance, and cognitive symptoms commonly referred to as “brain fog” or “fibro fog” [21,22]. Additionally, Martinez-Lavin and colleagues have hypothesized that fibromyalgia may often originate from peripheral nerve trauma, with neuropathic changes spreading to the dorsal root ganglia, leading to neuroplastic alterations and modified pain perception [23]. Despite these insights, the precise mechanisms underlying small fiber pathology in fibromyalgia remain incompletely understood, warranting further investigation [19].

Conversely, some evidence challenges the idea that fibromyalgia and SFN are linked, proposing instead that they represent distinct clinical entities. Nevertheless, it is widely acknowledged that there is frequent overlap, and many pharmacological and non-pharmacological treatments appear beneficial in both conditions [1,23].

Our retrospective study was grounded in the assumption that fibromyalgia shares pathophysiological aspects with SFN, positioning it as a complex syndrome of pain perception with challenging clinical and functional characteristics. In this retrospective analysis, patients were stratified into two groups based on self-reported symptoms suggestive of small fiber involvement and were re-evaluated after 12 weeks. Those treated with LAC and PEA as add-on therapy showed significant improvement in FIQR scores (−28%), reaching levels comparable to patients with milder symptoms who received SOC alone. This median reduction of 19 points in FIQR not only reflects a substantial numerical decrease but also exceeds the minimal clinically important difference (MCID) generally reported for this instrument, which averages around 14% of baseline scores based on previous studies [24]. This suggests that the observed improvement is not only statistically significant but also meaningful from the patient’s perspective.

In contrast, patients treated with SOC alone did not show significant change in FIQR scores. Baseline FIQR scores in this cohort were slightly, but not significantly, lower than those in the treatment group, making a ceiling effect unlikely. The absence of significant improvement may instead reflect a stabilization or plateau of symptoms under standard care, where patients maintain their condition without further deterioration or notable relief. This interpretation is consistent with the chronic and fluctuating nature of fibromyalgia, in which standard therapies may prevent symptom worsening but may not always induce substantial improvements.

The FIQR questionnaire assesses physical function across three domains: difficulty performing daily activities, general health status, and severity of disease symptoms. It is a validated and reliable tool widely used to measure functional capacity and disease severity in fibromyalgia, making it particularly suitable for assessing the impact of diffuse neuropathy and the effectiveness of LAC and PEA in this context [25].

Given that the role of peripheral neuropathy in fibromyalgia remains uncertain, it is challenging to interpret these results from a strictly pathophysiological perspective. One plausible explanation is that LAC and PEA attenuate the symptomatic contribution of peripheral nerve involvement within the overall fibromyalgia symptom complex. Alternatively, considering fibromyalgia’s multifaceted nature [26], these compounds may exert beneficial effects on the nervous system more broadly—whether peripheral, central, or both—resulting in improved symptomatology and quality of life. However, due to the observational and retrospective design of the study, pathophysiological interpretations should be considered with caution. Notably, our data show a significant shift from severe to moderate disease severity in patients receiving the combination treatment. While our findings are consistent with the involvement of peripheral small fibers in a subset of fibromyalgia patients, they do not establish causality. Controlled prospective studies with objective diagnostic measures are needed to confirm this hypothesis.

In designing our retrospective study, we excluded patients treated with anticonvulsants due to their established efficacy in various neuropathic pain conditions [27,28], aiming to better isolate the effects of LAC and PEA on fibromyalgia patients with suspected small fiber involvement. Interestingly, a recent randomized trial by Salaffi et al. demonstrated even greater FIQR improvements when LAC and PEA were added to duloxetine and pregabalin therapy in a more heterogeneous fibromyalgia population [15]. This suggests potential synergistic effects between pregabalin and the LAC+PEA combination, possibly through combined central and peripheral mechanisms.

Current treatments for fibromyalgia often provide limited relief, underscoring the need for novel, well-tolerated therapeutic options. Identifying clinical endotypes within fibromyalgia may facilitate more targeted interventions. Nutraceuticals and dietary approaches have shown promise in alleviating fibromyalgia symptoms, supporting further exploration of supplements such as LAC and PEA [8,29].

This study has several important limitations. First, due to its retrospective design, the study lacks randomization and is inherently prone to selection and information bias, although propensity score matching was used to partially address group imbalance. Second, the identification of patients with suspected small fiber involvement was based solely on clinical symptoms rather than objective diagnostic tests such as skin biopsy or corneal confocal microscopy, limiting the precision of phenotype classification. Third, the intervention was not limited to LAC and PEA alone, but was administered via a multi-compound nutraceutical (Kalanit^®^) that also included Boswellia serrata extract, vitamin B6, and vitamin E—substances with known anti-inflammatory and neuroprotective effects—which may confound attribution of clinical benefits [30,31,32]. Finally, the use of self-reported FIQR scores introduces the risk of response bias, particularly in a population with fluctuating symptoms and strong psychosomatic components.

## 5. Conclusions

Managing patients with fibromyalgia remains a significant challenge, especially for those who exhibit limited or no response to SOC. Our retrospective clinical experience supports the addition of LAC and PEA to SOC, demonstrating notable improvements in FIQR scores. This effect appears particularly relevant in patients with complex clinical presentations and/or symptoms suggestive of SFN, who often respond suboptimally to conventional fibromyalgia management.

These findings suggest that LAC and PEA, when used as adjunctive therapies, may offer a promising and effective option for improving outcomes in fibromyalgia patients with associated SFN. However, prospective controlled studies are necessary to confirm these results and to further clarify the mechanisms through which these compounds provide symptom relief in this challenging patient population.

## Data Availability

The data presented in this study are available upon request from the corresponding author, with permission from AOU Sant’Orsola-Malpighi (Bologna, Italy).

## Abbreviations

The following abbreviations are used in this manuscript:

ACR	American College of Rheumatology
CI	Confidence interval
FIQR	Fibromyalgia Impact Questionnaire Revised
FM	Fibromyalgia syndrome
IRB	Institutional Review Board
LAC	L-Acetyl Carnitine
PEA	Palmitoylethanolamide
PSM	Propensity score matching
SFN	Small fiber neuropathy
SOC	Standard of Care
